# Alignment between expanded carrier screening panels and conditions leading to PGT-M: a real-world cohort study

**DOI:** 10.1007/s10815-026-03992-1

**Published:** 2026-08-07

**Authors:** Einat Zivi, Avia Clymer, Orit Freireich, Gheona Altarescu, Ido Ben-Ami, Avi Tsafrir

**Affiliations:** 1 Maccabi Health Care Services, Sharon District, Israel; 2 Faculty of Medicine, Hebrew University of Jerusalem, Jerusalem, Israel; 3 PGT Unit, Medical Genetics Institute, Shaare Zedek Medical Center, Jerusalem, Israel; 4 IVF Unit, Department of Obstetrics and Gynecology, Shaare Zedek Medical Center, Jerusalem, Israel; 5 IVF Unit, Department of Obstetrics and Gynecology, Hadassah Medical Center, Jerusalem, Israel

**Keywords:** Expanded carrier screening, PGT-M, Reproductive genetics, Carrier screening, Autosomal recessive disorders, IVF

## Abstract

**Purpose:**

To assess the extent to which expanded carrier screening (ECS) panels capture the genetic conditions for which preimplantation genetic testing for monogenic diseases (PGT-M) is performed.

**Methods:**

A retrospective observational study conducted at a single university-affiliated PGT unit from 2015 to 2023. Women undergoing PGT-M for autosomal recessive or X-linked genetic conditions were included. Genetic conditions were cross-matched with five ECS panels (four commercial panels and one publicly funded ECS panel) and compared with a targeted, population-specific screening. Coverage was assessed at both the woman and gene levels using paired statistical comparisons.

**Results:**

A total of 330 cases were included; seven women underwent PGT-M for two eligible conditions and were therefore counted twice. These cases involved 110 different genes with an uneven distribution: eight genes accounted for 53.2% of cases, while 71 genes were observed in a single case. At the individual-woman level, targeted, population-specific screening showed lower coverage (47.9%) than that of all ECS panels (71.8–91.5%), with overall differences statistically significant (*P* < 0.001). At the gene level, coverage ranged from 20.9 to 79.1% across panels (*P* < 0.001). The evaluated ECS panels included 271–787 genes, with the largest panel demonstrating higher case coverage than the other commercial panels.

**Conclusions:**

Expanded carrier screening panels capture most autosomal recessive and X-linked genetic conditions for which PGT-M is performed. These findings support the clinical utility of ECS in identifying couples at risk of having children affected by such conditions.

**Supplementary Information:**

The online version contains supplementary material available at https://doi.org/10.1007/s10815-026-03992-1.

## Introduction

More than 2000 genes are associated with childhood-onset autosomal recessive (AR) or X-linked (XL) genetic conditions [1]. Historically, carrier status was identified only after the birth of an affected child. Early programs focused on ethnicity-based screening, targeting high-prevalence disorders within specific populations such as Tay-Sachs disease in Ashkenazi Jews [2] and β-thalassemia in Mediterranean countries [3], followed by “pan-ethnic” screening for conditions like cystic fibrosis in the general population [4]. Subsequently, several countries implemented screening for genetic conditions above defined frequency thresholds [5].

With the advent of next-generation sequencing (NGS) and the decline in sequencing costs, carrier screening has advanced into the era of ECS panels, enabling the simultaneous assessment of hundreds of genes associated with severe recessive and X-linked disorders. ECS is now widely available through commercial providers and is offered either before conception or during pregnancy [6]. Large-scale studies have shown that 1.9–2.6% of reproductive couples are newly identified as having an increased risk of having a child affected by one of these conditions [7–10]. However, it remains unclear to what extent ECS panel content aligns with the genetic conditions for which PGT-M is performed in clinical practice.

At-risk carrier couples who wish to avoid the birth of an affected child may consider several reproductive options: in vitro fertilization (IVF) with PGT-M, which enables transfer of unaffected embryos, or natural conception followed by invasive prenatal diagnosis and selective termination if the fetus is affected.

Current panels include genes for conditions that some couples may not consider severe enough to warrant PGT-M, such as hemochromatosis, or for diseases with effective treatments, such as Gaucher disease. However, it remains unclear whether ECS panel content reflects the conditions that actually lead couples to pursue PGT-M in clinical practice. Couples who undergo PGT-M represent a unique subgroup: they have bee identified as carriers of a condition they consider sufficiently serious to justify the procedure. This population, therefore, provides an opportunity to assess which conditions detected through preconception carrier screening lead to PGT-M in clinical practice. In this study, we evaluated how well representative ECS panels cover  the genetic conditions for which PGT-M was performed in a large PGT unit.

## Methods

This was an observational, descriptive study based on a review of medical records at a single, university-affiliated public medical center. The study population included couples and single women who underwent oocyte retrieval for PGT-M due to carrier status for AR or XL conditions. The study was approved by the Shaare Zedek Medical Center Institutional Review Board (approval SZMC0051-24).

In Israel, all citizens are covered by a national health insurance plan that provides access to pregnancy-related genetic testing. Among these services, a national ethnicity-based carrier screening program, funded by the Ministry of Health, has been available since 2002. This includes severe, pediatric-onset, untreatable diseases with a carrier frequency ≥ 1:60 or a disease frequency ≥ 1:15,000. Approved conditions are funded through the national health basket, with optional second-tier panels available through health maintenance organizations. In November 2024, this ethnicity-based panel was replaced by a publicly funded pan-ethnic ECS panel including 292 genes. During the study period, citizens were eligible for the publicly funded ethnicity-based panel and could also opt for private, commercial ECS testing. PGT-M in Israel is publicly covered for couples at risk of transmitting severe genetic diseases.

## Data collection

We reviewed the medical records of all women who underwent oocyte retrieval for PGT and classified the cases by indication. We then identified women who underwent PGT-M for autosomal recessive or X-linked conditions that ECS panels could potentially detect. We excluded PGT-M cases performed for dominant or digenic inheritance, sub-chromosomal deletions or duplications, and Rh incompatibility, as these do not fall within the scope of reproductive ECS.

We compared coverage across four commercial ECS panels and one publicly funded pan-ethnic ECS panel. Additionally, we evaluated a publicly funded ethnicity-based carrier screening method used in Israel during the study period. Unlike ECS panels, the ethnicity-based method is not a fixed gene panel but a population-specific set of targeted tests determined by reported ancestry.

We chose four representative commercial ECS panels commonly used in clinical practice, hereafter referred to as Panels 1–4 for ease of presentation without implying ranking: panel 1 (Beacon 787, Fulgent Genetics; 787 genes), panel 2 (CentoScreen Paired, Centogene; 333 genes), panel 3 (Foresight, Myriad Women’s Health; 271 genes), and panel 4 (Horizon 421, Natera; 421 genes), based on publicly available gene lists. These panels were selected based on widespread clinical use and the availability of publicly accessible gene lists at the time of data collection. Thus, the comparison reflects representative versions in use during the study period and is not intended as a formal comparative evaluation.

For patients who underwent PGT-M for two conditions, if only one was autosomal recessive or X-linked (e.g., the second was autosomal dominant), the woman was counted as one case. If both conditions were autosomal recessive or X-linked, patients tested for both conditions were counted as two cases.

## Statistics

### Design and outcome definition

Two paired binary datasets were analyzed.

1. Women-level dataset (*N* = 330 cases)

Each woman was classified as covered (1) or not covered (0) by each screening panel. Outcomes were paired within the same woman across panels.

2. Gene-level dataset (*N* = 110 genes).

Each gene was classified as detected (1) or not detected (0) for each screening panel. Outcomes were paired across panels within the same gene.

### Descriptive statistics

For each panel, coverage  rates were calculated as proportions and reported with 95% confidence intervals (CIs) using Wilson’s method.

### Inferential statistics

To test for overall differences across the six screening approaches under a within-unit (paired) design, we applied Cochran’s *Q* test.

Post hoc pairwise comparisons were conducted using exact McNemar tests.

To correct for multiple comparisons, *P*-values were adjusted using the Holm step-down procedure.

All tests were two-sided, with Holm-adjusted *P* < 0.05 defining statistical significance.

## Results

### Cohort description

**Fig. 1:**
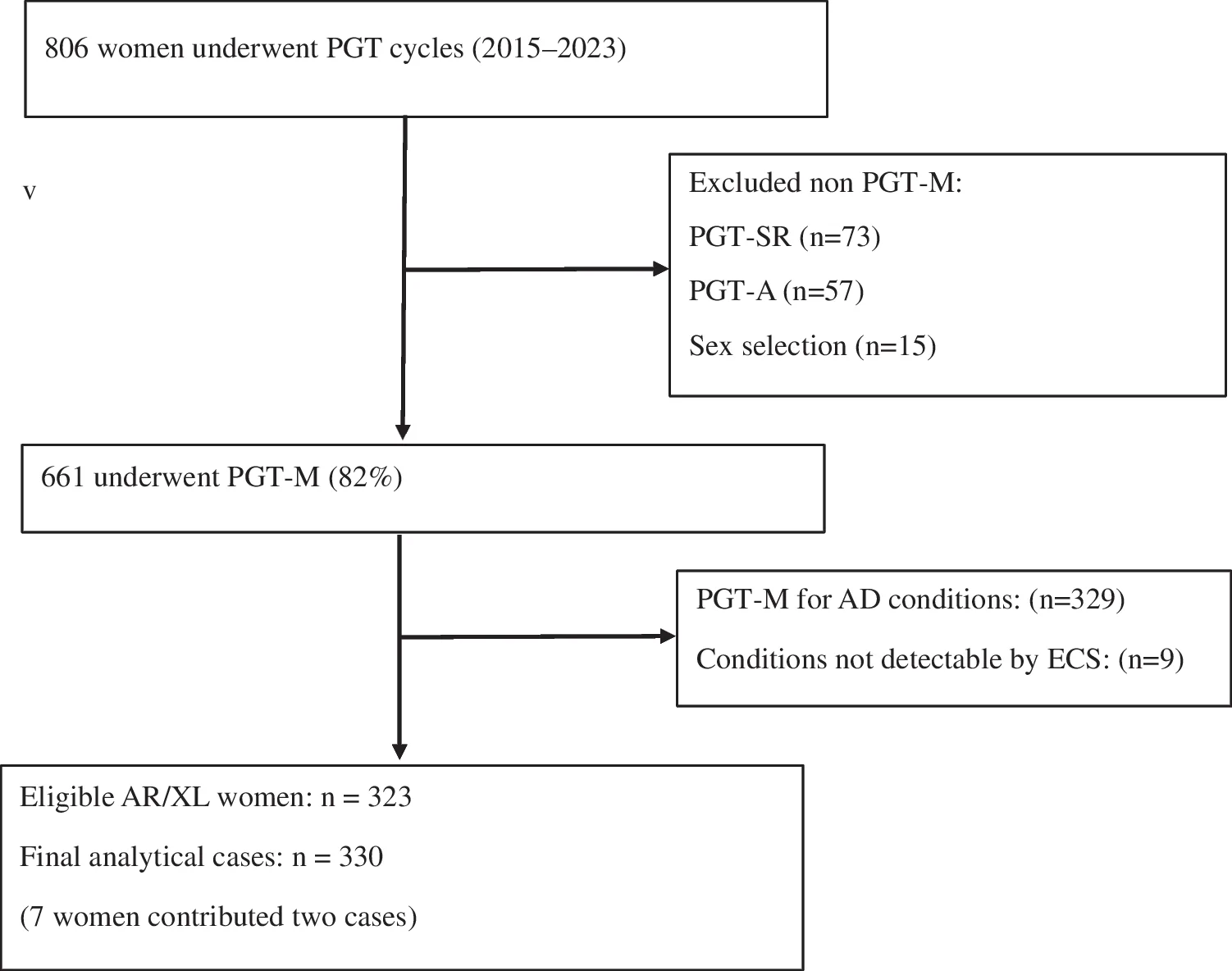
Flowchart of study cohort selection for the PGT-M analysis.  AD, autosomal dominant; AR, autosomal recessive; XL, X-linked; ECS, expanded carrier screening; PGT-M, preimplantation genetic testing for monogenic disorders; PGT-SR, preimplantation genetic testing for chromosomal structural rearrangements; PGT-A, preimplantation genetic testing for aneuploidy.

A total of 806 women underwent at least one oocyte retrieval procedure for PGT between 2015 and 2023. Of these, 661 (82.0%) underwent PGT-M (Fig. 1). Nearly half of all PGT-M cases (*n* = 338, 49%) involved autosomal dominant conditions (*n* = 329) or other genetic conditions not detectable by ECS, such as Rh incompatibility, chromosomal deletions or duplications, or complex inheritance patterns (*n* = 9). These cases were excluded from the present analysis, leaving 323 women (48.9%) in the study cohort. Seven women underwent PGT-M for two eligible conditions and were therefore counted twice for analytical purposes, yielding 330 cases. Demographic and clinical characteristics of the study cohort are presented in Table 1.

**Table 1:** Demographic and clinical characteristics of women undergoing pgt-m for autosomal recessive or X-linked conditions (*N* = 323)

Characteristic	Value
Age at PGT-M (years), mean ± SD	30.4 ± 5.2
Number of children, mean ± SD	1.56 ± 1.58
No children, %	33.2
One child, %	22.4
Two children, %	20.2
Three or more children, %	23.6
Previous child affected by the tested condition, %	46.5
Previous pregnancy termination due to the tested condition, %	15.7
Infertility, %	14.5
Mode of inheritance, %	
Autosomal recessive (AR)	59.5
X-linked (XL)	40.5
Maternal population background, %	
Ashkenazi Jewish	38.3
Mizrahi Jewish	20.7
Arab	14.2
Mixed Jewish	8.9
Unknown	17.9
Paternal population background, %	
Ashkenazi Jewish	37.7
Mizrahi Jewish	20.4
Arab	13.6
Mixed Jewish	8.8
Unknown	9.5

Population background was self-reported. Ashkenazi and Mizrahi refer to Jewish population subgroups as commonly classified in Israeli clinical practice. “Mixed” indicates mixed Jewish background; “unknown” indicates missing data. Values are presented as mean ± standard deviation for continuous variables and percentages for categorical variables

### Distribution of genetic conditions for which PGT-M was performed

**Fig. 2:**
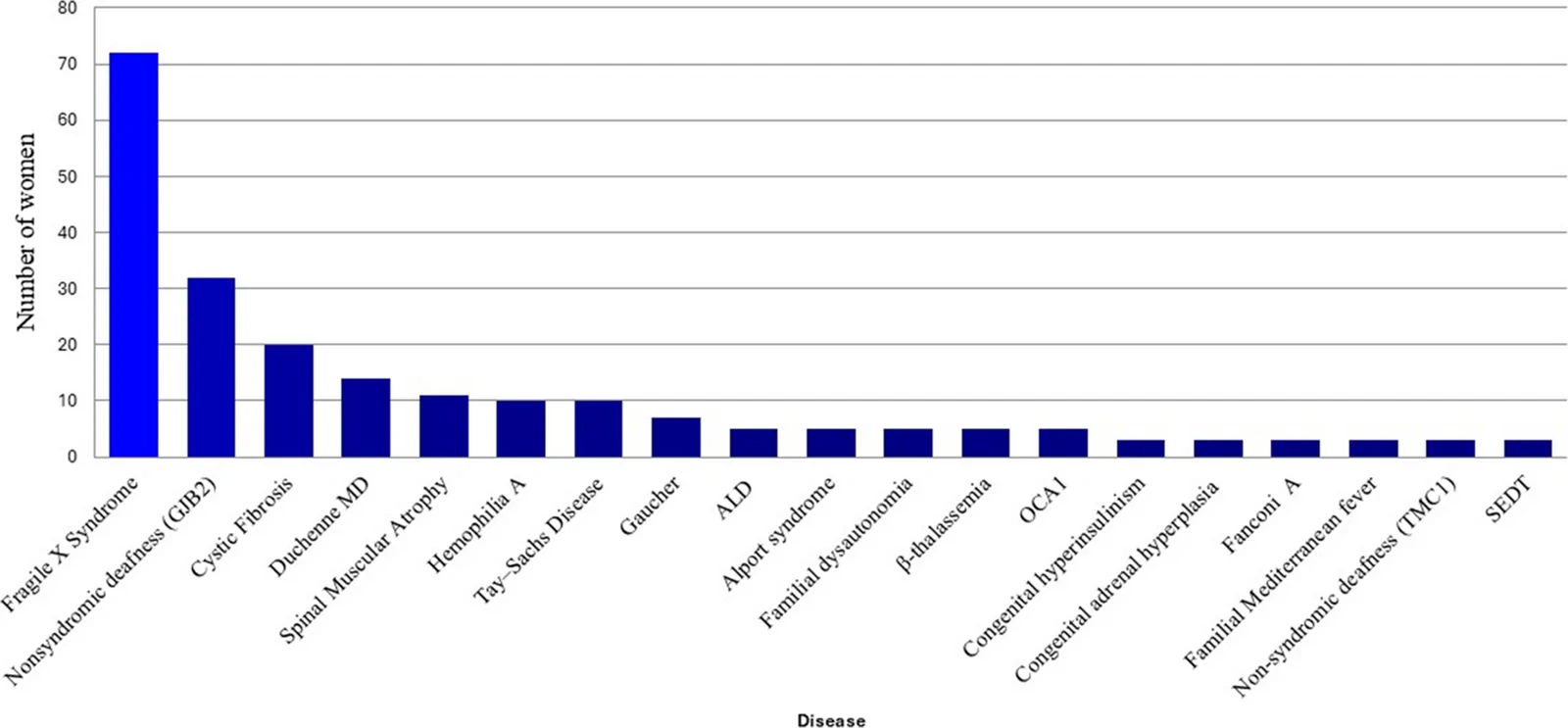
Distribution of diseases for which PGT-M was performed (diseases with ≥ 3 cases shown). Have been covered by different reproductive carrier screening panels. ALD, X-linked adrenoleukodystrophy; OCA1, oculocutaneous albinism type 1; TMC1, transmembrane channel-like 1; SEDT, spondyloepiphyseal dysplasia tarda.

Patients in this cohort underwent PGT-M due to pathogenic variants spanning 110 distinct genes. The distribution of genes was uneven: the eight most frequently tested genes accounted for 53.2% of all PGT-M cases (Fig. 2). In contrast, 20 genes were observed in two women each, while 71 genes were observed in only one woman (Supplementary Table S1).

### Women-level comparison

**Fig. 3:**
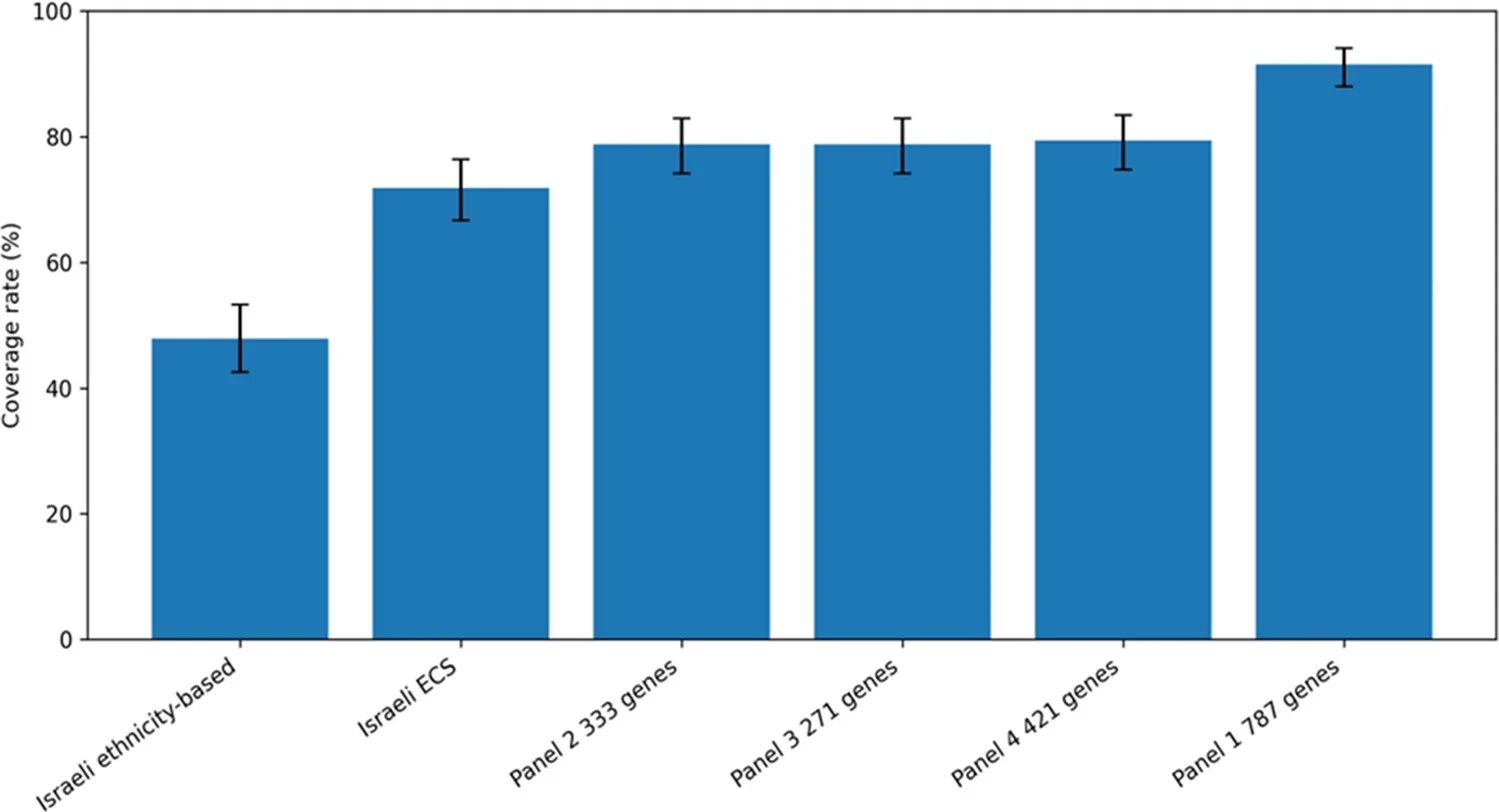
Proportion of women undergoing PGT-M whose mutated genes would have been covered by different reproductive carrier screening panels. Note: Error bars indicate 95% Wilson score confidence intervals. Positivity was defined as a woman having at least one mutated gene covered by the respective panel. *N* = 330 women

Case-level analysis (330 PGT-M cases) showed variation in panel coverage (Cochran’s *Q*(5) = 319.77, *P* < 0.001). Coverage rates varied across screening panels (Table 2). The Israeli ethnicity-based panel classified 47.9% of women as positive (95% CI [42.5–53.3]), whereas the largest commercial ECS panel (panel 1, 787 genes) classified 91.5% as positive (95% CI [88.0–94.1]). The mid-range commercial panels showed similar coverage rates (78.8–79.4%), while the Israeli ECS panel (292 genes) identified 71.8% of women. Figure 3 provides a visual comparison of coverage across panels.

**Table 2:** Coverage of women’s PGT-M conditions across screening approaches. Coverage represents the proportion of women whose PGT-M condition would have been included in each screening approach (*N* = 330 cases)

Screening approach	Women covered (*n*)	Coverage rate (%)	95% CI (Wilson)
Israeli ethnicity–based screening method	158	47.9	42.5–53.3
Publicly funded ECS panel	237	71.8	66.7–76.4
Panel 1, 787 genes	302	91.5	88.0–94.1
Panel 2, 333 genes	260	78.8	74.1–82.9
Panel 3, 271 genes	260	78.8	74.1–82.9
Panel 4, 421 genes	262	79.4	74.7–83.4

Pairwise McNemar tests with Holm correction showed that panel 1 identified significantly more women than all other panels, whereas the ethnicity-based panel identified significantly fewer women than all other panels. Differences among the mid-range commercial panels were not statistically significant (Supplementary Table 2).

Nearly half of the women in the study cohort had previously given birth to a child affected by the condition for which they later underwent PGT-M. In this subgroup, the corresponding ECS panel coverage ranged from 66.0 to 88.9%.

### Gene-level comparison

**Fig. 4:**
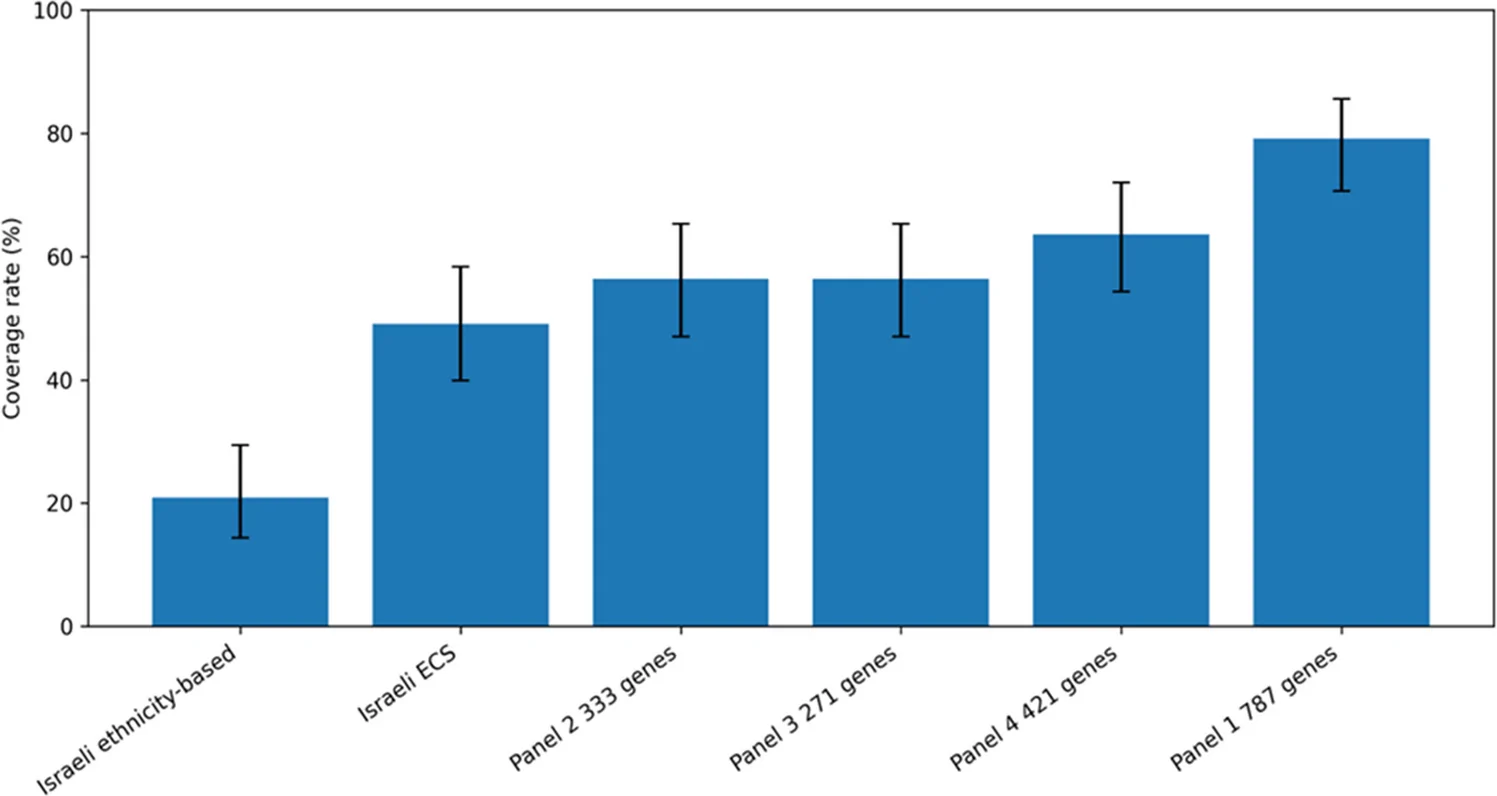
Coverage of PGT-M genes across reproductive genetic screening panels. Note: Bars represent the proportion of women whose mutated gene(s) would have been covered by each reproductive carrier screening panel. Error bars indicate 95% Wilson score confidence intervals. Positivity was defined as a woman having at least one mutated gene covered by the respective panel. *N* = 330 women

Gene-level analysis (*N* = 110 genes) demonstrated variation in panel coverage (Cochran’s *Q*(5) = 130.82, *P* < 0.001). Coverage ranged from 20.9% for the ethnicity-based screening to 79.1% for the largest commercial panel (Table 3), with intermediate panels covering 49–64% of genes. Figure 4 provides a graphical comparison of gene-level coverage across panels.

**Table 3:** Coverage of PGT-M genes across screening approaches. Coverage represents the proportion of unique PGT-M genes included in each screening approach (*N* = 110)

Screening approach	Genes covered (*n*)	Coverage (%)	95% CI (Wilson)
Israeli ethnicity–based screening method	23	20.9	14.4–29.4
Publicly funded ECS panel	54	49.1	39.9–58.3
Panel 1, 787 genes	87	79.1	70.6–85.6
Panel 2, 333 genes	62	56.4	47.0–65.3
Panel 3, 271 genes	62	56.4	47.0–65.3
Panel 4, 421 genes	70	63.6	54.3–72.0

The genes not covered by any expanded carrier screening panel were almost entirely those represented by a single case in the cohort (17 genes, 21 cases [6.4%], of which 14 were single-case), consistent with the gap between woman-level and gene-level coverage (Supplementary Table S1). Three uncovered genes had more than one case. STRC (*n* = 2) is recognized as a technical limitation of sequencing-based panels, owing to its pseudogene and predominantly copy-number mechanism, while TRAPPC2 (*n* = 3) and CACNA1F (*n* = 2) cause milder conditions outside the severe, early-onset scope these panels typically target.

Pairwise McNemar comparisons yielded a similar pattern, with the largest panel covering significantly more genes than all other panels and the ethnicity-based panel covering significantly fewer genes than the remaining panels (Supplementary Table 3).

## Discussion

ECS panels captured a substantial proportion of the genetic conditions for which PGT-M was performed, indicating alignment between panel content and the spectrum of conditions observed in this cohort. PGT-M utilization in this setting reflects clinical practice rather than directly measuring disease severity.

Most evaluations of expanded carrier screening panels are based on carrier frequencies, modeled populations, or theoretical estimates of at-risk couples. In contrast, the present analysis evaluates panel coverage against the genetic conditions for which PGT-M was performed. This approach provides a complementary perspective by linking panel content to observed PGT-M utilization, which should not be interpreted as a direct measure of disease severity or clinical prioritization.

Two prior studies from Israel have shown that ECS identifies substantially more at-risk couples than ethnicity-based methods [8, 10]. Our results are consistent with these findings. Furthermore, broader ECS panels provided greater coverage of the conditions for which PGT-M was performed. The American College of Medical Genetics and Genomics has proposed a tiered framework for gene selection, recommending a tier-3 panel of 113 genes for screening before or in early pregnancy [11]. In this analysis, the commercial panels examined included approximately 270–780 genes and would likely have identified a larger proportion of the pathogenic variants observed in this cohort. Although several conditions were relatively more frequent, limiting screening to only the most common conditions would have missed a meaningful number of at-risk couples.

In this cohort, nonsyndromic hearing loss was among the most frequent indications for PGT-M. While some screening tests have chosen not to include genes associated with this condition [1], it is included in all commercial ECS panels examined in this study. This finding indicates that, in this setting, conditions not universally prioritized in screening policy may nonetheless be associated with PGT-M utilization.

The strengths of this study include its focus on couples who underwent PGT-M and its relatively large sample size for this clinical context.

Several limitations should be acknowledged. First, the analysis was restricted to autosomal recessive and X-linked conditions and therefore does not reflect the full spectrum of indications for PGT-M. Second, the analysis includes only conditions for which PGT-M was performed and does not capture the full spectrum of carrier couples identified through screening, nor the proportion of at-risk couples who did not pursue PGT-M. Third, information on how carrier status was initially established was not consistently available, precluding analysis of whether couples were identified through ECS, other screening programs, family history, or the birth of an affected child. Fourth, this was a single-center study conducted within a publicly funded healthcare system that provides broad access to IVF and PGT-M. The distribution of conditions observed in this cohort likely reflects not only underlying genetic epidemiology but also the high accessibility and utilization of PGT-M within the Israeli healthcare system, which may differ from other settings. Fifth, the screening approaches evaluated differ methodologically: the Israeli publicly funded ethnicity-based program is based on targeted testing of specific variants, whereas the commercial panels rely on sequencing-based approaches. For the targeted program in particular, inclusion of a gene does not ensure that the specific causative variant would have been tested, so its coverage as reported here may be overestimated relative to the sequencing-based panels. Sixth, panel coverage was assessed by gene inclusion and does not account for whether a specific variant would be reported, as sequencing-based panels do not report variants of uncertain significance; the coverage rates are therefore an upper bound. This mainly concerns the rarer conditions, since the most frequent ones, such as Fragile X and cystic fibrosis, are detected by methods not affected by this limitation, so its impact on the woman-level estimates is small. Seventh, panel content was assessed as of data collection; as ECS panels expand over time, coverage relative to current content may modestly exceed that available in earlier years.

In conclusion, this study shows that a substantial majority of the autosomal recessive and X-linked conditions for which couples underwent PGT-M are detectable by expanded carrier screening. ECS before pregnancy may facilitate earlier identification of at-risk couples who may consider PGT-M. These findings have direct implications for genetic counseling, preconception care, and the design and implementation of screening policy.

## Supplementary information

Below is the link to the electronic supplementary material.

Supplementary file1 (XLSX 19 kb)

Supplementary file2 (DOCX 15 kb)

Supplementary file3 (DOCX 16 kb)

## Data Availability

No datasets were generated or analysed during the current study.
