# Supplementary material for: Alignment between expanded carrier screening panels and conditions leading to PGT-M: a real-world cohort study
Source: J Assist Reprod Genet. 2026 Aug 7;43(9):2963–70. doi: 10.1007/s10815-026-03992-1 (PMC13731626; doi:10.1007/s10815-026-03992-1)
Supplement: Supplementary file 1 [file 10815_2026_3992_MOESM2_ESM.docx]

Supplementary Table 2. Women-level pairwise differences between carrier screening approaches (exact McNemar tests, Holm-adjusted). ✓ indicates Holm-adjusted P < 0.05; pairwise comparisons were performed using exact McNemar tests on paired binary outcomes across women.

| approach | Israeli ethnicity-based panel | Israeli ECS panel | Panel 1, 787 genes | Panel 2,  333 genes | Panel 3, 271 genes | Panel 4,  421 genes |
| --- | --- | --- | --- | --- | --- | --- |
| Israeli ethnicity-based panel | - | ✓ | ✓ | ✓ | ✓ | ✓ |
| Israeli ECS panel |  | - | ✓ |  | ✓ | ✓ |
| Panel 1, 787 genes |  |  | - | ✓ | ✓ | ✓ |
| Panel 2, 333 genes |  |  |  | - |  |  |
| Panel 3, 271 genes |  |  |  |  | - |  |
| Panel 4, 421 genes |  |  |  |  |  | - |
